# Supplementary material for: Systematic analysis of NAC transcription factors in Gossypium barbadense uncovers their roles in response to Verticillium wilt
Source: PeerJ. 2019 Nov 5;7:e7995. doi: 10.7717/peerj.7995 (PMC6839521; doi:10.7717/peerj.7995)
Supplement: Figure S1 [file peerj-07-7995-s001.pdf]

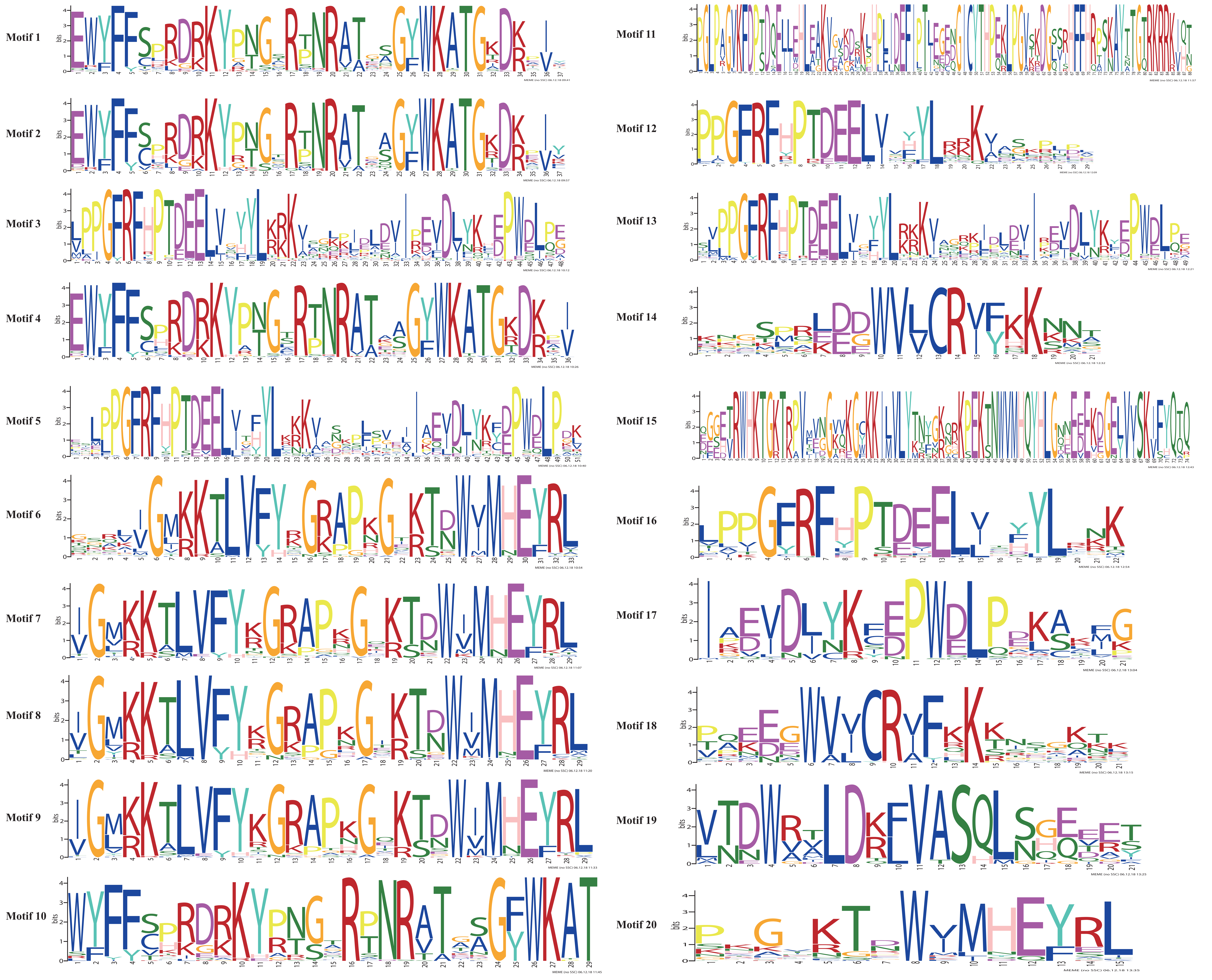
Motif 1
Motif 2
Motif 3
Motif 4
Motif 5
Motif 6
Motif 7
Motif 8
Motif 9
Motif 10
Motif 11
Motif 12
Motif 13
Motif 14
Motif 15
Motif 16
Motif 17
Motif 18
Motif 19
Motif 20
