# Supplementary figures and images for: Systematic analysis of NAC transcription factors in Gossypium barbadense uncovers their roles in response to Verticillium wilt
Source: PeerJ. 2019 Nov 5;7:e7995. doi: 10.7717/peerj.7995 (PMC6839521; doi:10.7717/peerj.7995)

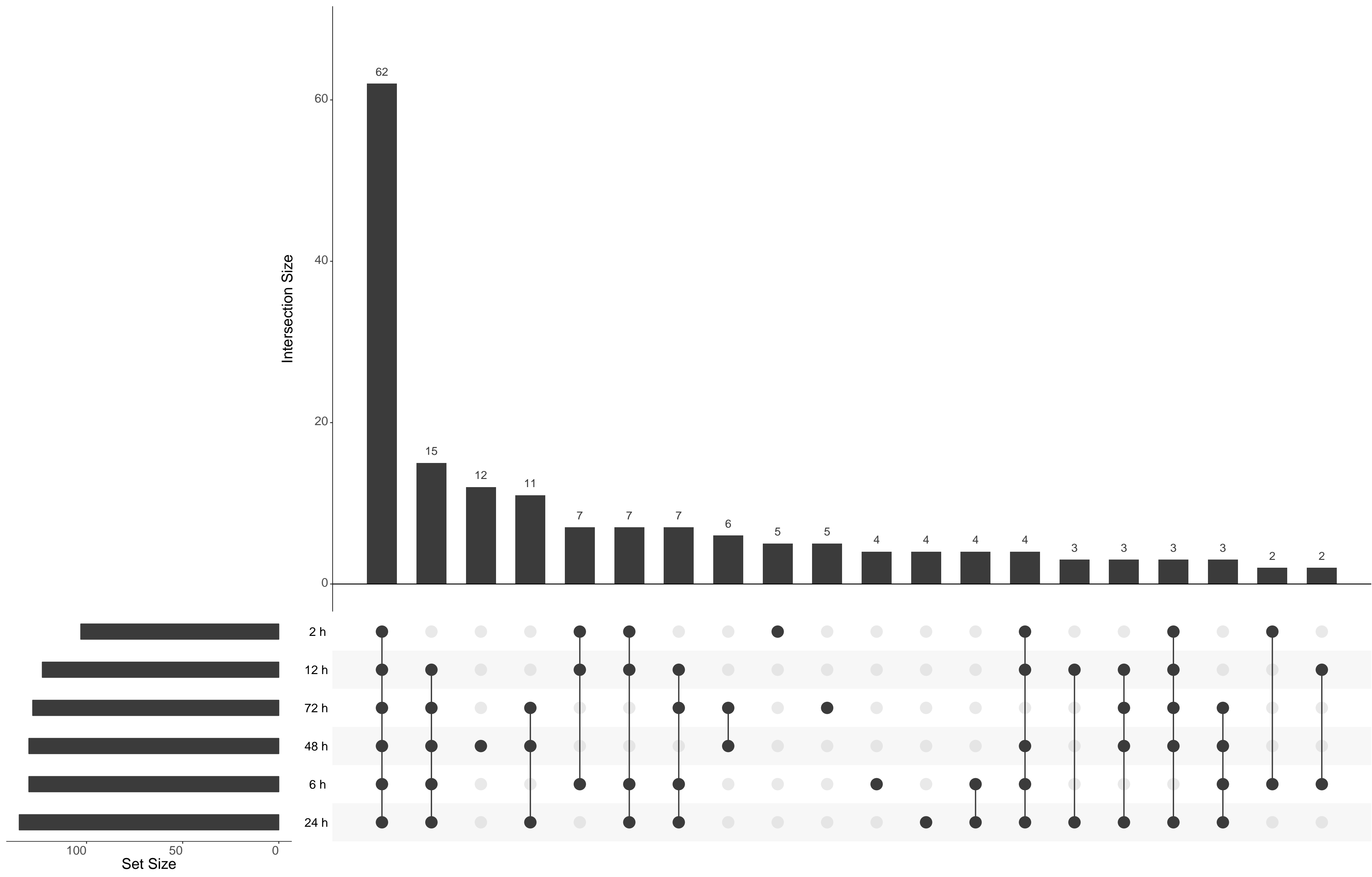

Supplement: Figure S2 — The online software jvenn was used to construct the Venn map of differentially expressed GbNAC genes. The number indicates the specific genes expressed in one time point or shared genes expressed in 2 to 6 time point. [file peerj-07-7995-s002.pdf]

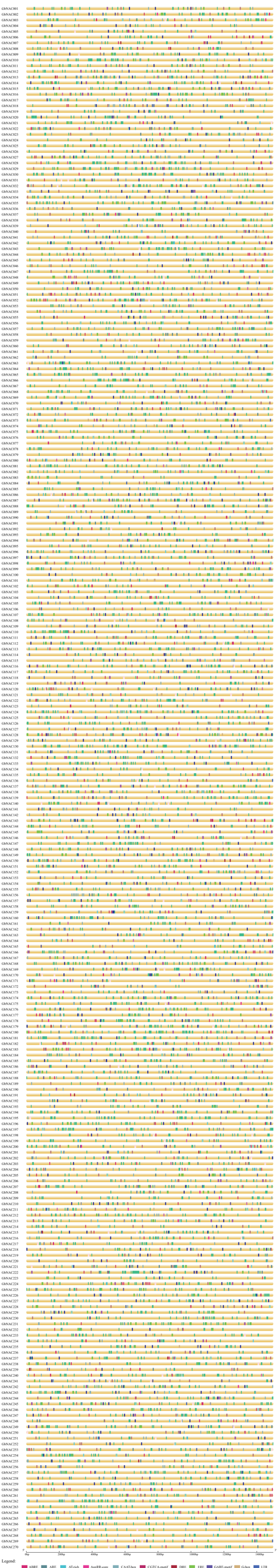

Supplement: Figure S3 — The scale bar at the bottom denotes the length of promoter sequence. [file peerj-07-7995-s003.pdf]
